# The HER2 flip-HER2 amplification of tumor cells in the cerebrospinal fluid of breast cancer patients with leptomeningeal disease: implications for treating the LM tumor with anti-HER2 therapy

**DOI:** 10.3389/fonc.2024.1402651

**Published:** 2024-05-17

**Authors:** Priya U. Kumthekar, Barbara Blouw, Perry Corkos, Seema Nagpal, Arushi Tripathy, David Piccioni, Michael Youssef

**Affiliations:** ^1^ Neurology (Neuro-Oncology) and Medicine (Hematology and Oncology), Northwestern Medicine Lou and Jean Malnati Brain Tumor Institute, Chicago, IL, United States; ^2^ Biocept, San Diego, CA, United States; ^3^ Neurology and Neurological Sciences, Neurosurgery, Stanford University Medicine, Stanford, CA, United States; ^4^ Neurosurgery, Michigan University Medicine, Ann Arbor, MI, United States; ^5^ Neurosciences, Neuro-Oncology, University of San Diego Health, San Diego, CA, United States; ^6^ Department of Neurology and Hematology, University of Texas Southwestern Medical Center, Dallas, TX, United States

**Keywords:** breast cancer, leptomeningeal disease, cerebrospinal fluid, CNSide, HER2

## Abstract

**Introduction:**

CNSide is a platform that detects and characterizes tumor cells in the cerebrospinal fluid (CSF) of patients with leptomeningeal disease (LMD). The platform was validated per College of American Pathologists (CAP) and Clinical Laboratories Improvement Amendment (CLIA) guidelines and run as a commercial Laboratory Developed Test (LDT) at Biocept in San Diego, CA. The platform allows CSF tumor cell (CSF-TC) enumeration and biomarker characterization by fluorescent *in situ* hybridization (FISH).

**Methods:**

We performed a multicenter retrospective chart review of HER2 FISH CNSide test results that were commercially ordered on 26 patients by physicians for LMD breast cancer patients between April 2020 and October 2022.

**Results:**

We show that HER2 is amplified on CSF tumor cells in 62% (16/26) of LMD breast cancer patients. 10/26 (38%) patients had discordant HER2-positivity between the primary tumor tissue and CSF-TC; of these, 35% (9/26) of the patients displayed HER2 amplification on the CSF-TCs, however were categorized as HER2 negative on the primary tumor. Of the 27% (7/26) patients with a HER2 positive primary tumor, one patient showed a HER2 negative LMD tumor. Two patients, 8% (2/26) had a HER2 equivocal primary tumor; of these, one demonstrated a HER2 negative, and one a HER2 positive LMD tumor. Serial analysis (at least 4 longitudinal tests) of HER2 status of the CSF-TC throughout therapy was available for 14 patients and demonstrated that HER2 status of the LMD changed in 29% (4/14) during their treatment course and impacted care decisions.

**Conclusions:**

Our data suggests that CSF-TC HER2 FISH analysis in LMD breast cancer patients may be discordant to the primary tumor sample and the discovery of HER2 positivity in the CSF may open doors to anti-HER2 targeted therapy options for LMD patients.

## 1 Introduction

Leptomeningeal Disease (LMD) or seeding of tumor cells to the pia and arachnoid mater, is a devastating complication of patients who have cancer. The incidence of LMD varies by cancer origin, from an incidence of 3-4% in patients having Non-Small Cell Lung Cancer (NSCLC) (1), to 1-8% of all breast cancers (2–4). However, autopsy results suggest LMD is underdiagnosed (5, 6) and actual incidence is far higher. Survival for patients with breast cancer LMD is dismal with an overall survival (OS) ranging from 3-11 months from diagnosis (1, 7).The diagnosis and treatment of LMD is constrained by poor sensitivity and reliability of the current standard of care diagnostic modalities including MRI imaging, CSF cytology and evaluation of clinical symptoms. This diagnostic challenge makes the assessment of treatment response and the designing of effective clinical trials in LMD challenging.

Treatment options for patients having LMD aim to prolong survival and to alleviate symptoms (8–10). Therapeutic strategies include whole brain radiation therapy (WBRT), craniospinal irradiation (CSI), intrathecal therapy (IT), and systemic therapy that penetrates the blood brain barrier (BBB) and blood CSF barrier (BCSFB). The goal of treatment should be to improve, or at least stabilize, neurological function, as well as improve OS without compromising quality of life (QOL).

RT has remained a mainstay in LMD treatment for decades. Various RT modalities are listed in the National Comprehensive Cancer Network (NCCN) guidelines including proton CSI, photon involved field radiation therapy (IFRT), WBRT as well as stereotactic radiosurgery (SRS). Systemic therapies have historically been less favorable for brain metastases and LMD given overall poor BBB/BCSFB penetrance. However, particularly for HER2 positive breast cancer LMD, novel systemic HER2-directed therapies with good CSF penetrance have been identified, including drugs such as tucatinib and trastuzumab deruxtecan (11, 12). Intrathecal targeted agents have recently also shown benefit. Kumthekar et al. demonstrated that intrathecal trastuzumab in HER2+ LMD breast cancer patients led to improved OS of 10.5 months (13). Despite these advances, there is no standard of care treatment for patients diagnosed with LMD. In fact, given the prognosis, in patients with a low Karnofsky performance status (<60%), with multiple, serious, or major neurological deficits, extensive systemic disease, and bulky central nervous system disease, a palliative/supportive care approach is commonly recommended (14, 15). It has been previously demonstrated that specific mutational status can be different in the primary vs metastatic vs brain metastatic tumor (16). Given ongoing discovery of novel therapeutic targets against specific mutations, going forward it will be critical to identify brain- and LMD-specific mutational status to optimize treatments for patients with brain metastases.

Challenges in evaluating the response to therapy of the LMD tumor have hampered clinical trial development for patients with LMD. This has led to exploration of additional methodologies, including the detection and quantification of CSF-TCs using CellSearch™, and CSF derived cell-free tumor DNA (cfDNA). CellSearch is FDA cleared for the detection of circulating tumor cells in blood and was shown to have improved sensitivity and specificity compared to cytology in a prospective study performed in LMD patients with epithelial tumor origin (17, 18). CellSearch was also used to demonstrate that changes in CSF-TC count during anti-HER2 directed therapy were associated with response to therapy in a prospective study of LMD breast cancer patients treated with IT trastuzumab (19). However, the CellSearch platform has limited utility: detection is restricted to CSF-TCs expressing epithelial cell adhesion molecule (EpCAM) and is therefore applicable only to patients with melanoma LMD. Furthermore, CellSearch may exclude the critical epithelial to mesenchymal transition (EMT) seen in LMD and has an upper limit of detecting 200 cells per sample (20).

CNSide™ (Biocept, San Diego) is a CAP/CLIA validated commercially run platform that quantifies and characterizes CSF-TCs. Recently using the CNSide platform, it was suggested that change in CSF-TC density corresponds with clinical response to therapy and can help with the detection of actionable biomarkers in the CSF (21). Here we describe a retrospective chart review of 26 unique LMD breast cancer patients treated at Northwestern University and University of Texas Southwestern Medical Center, in whom CNSide testing was utilized to analyze CSF for tumor cell number and HER2 status.

## 2 Materials and methods

### 2.1 Patient demographics and CSF collection

**Figure 1:**
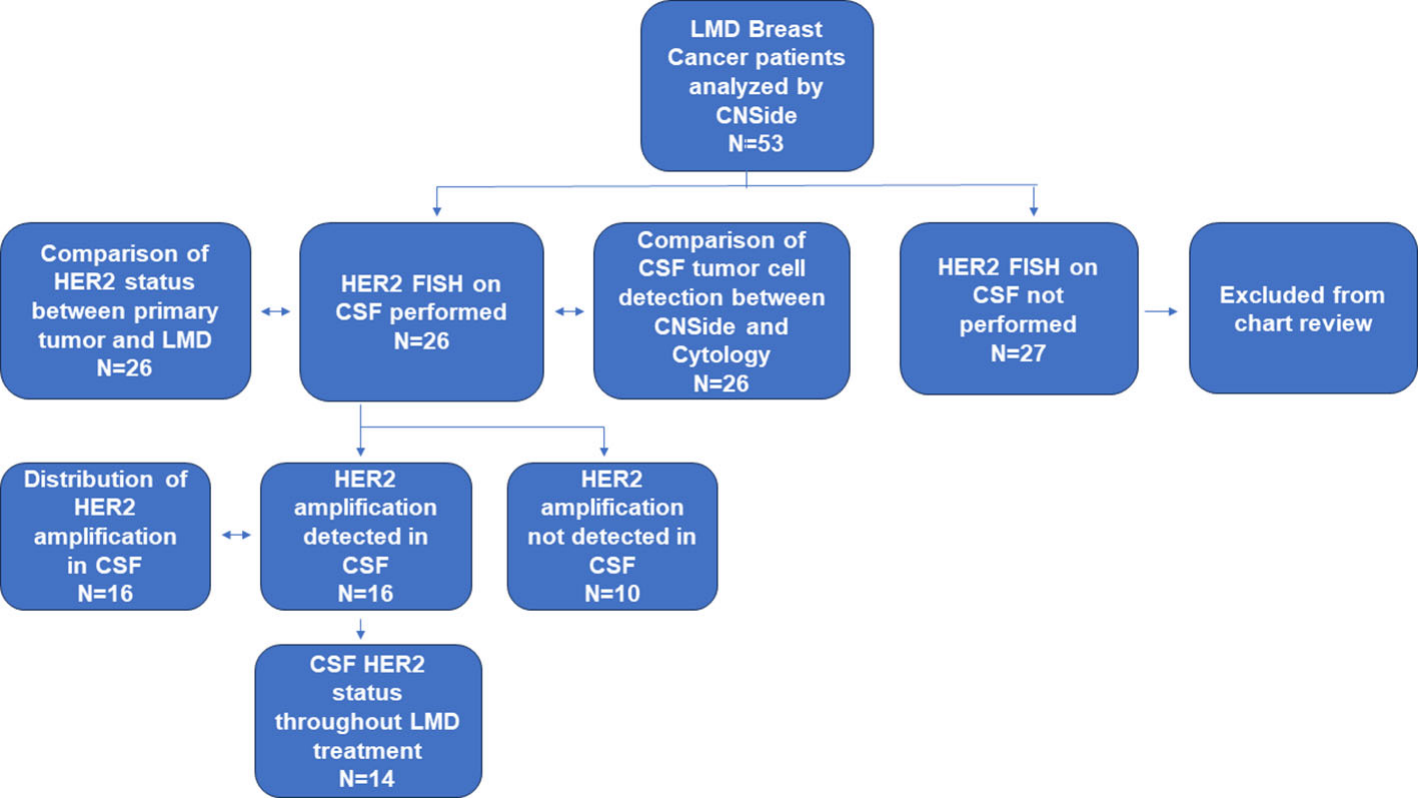
Cohort and analysis performed. In this cohort, the CSF of 53 patients having breast cancer and confirmed LMD was analyzed by CNSide. The CSF of 26 these patients was tested for HER2 FISH. In this group, the HER2 status of the CSF was compared to that of the primary tumor, and CSF tumor cell detection between conventional cytology and CNSide was compared, respectively. HER2 was amplified in the CSF in 16 patients, which were further analyzed for distribution of HER2 amplification. For 14 patients the HER2 status of the CSF was evaluated throughout their LMD treatment.

Data was collected on patients from both the Northwestern Medicine Lou and Jean Malnati Brain Tumor Institute, as well as at the University of Texas Southwestern Medical Center under institutional review board (IRB) approved retrospective protocols. The informed consent requirement was waived by the respective IRBs as most patients were no longer alive during time of analysis or were lost to follow up. Patients included were those who had breast cancer, with a suspected or confirmed diagnosis of LMD. CSF was collected at timepoints in line with standard of care in the diagnosis and treatment of patients with LMD. CSF was collected in institutional provided collection tubes; a portion of the CSF was analyzed for cytology at each institution’s respective histopathology laboratory and evaluated for presence of tumor cells by institutional board-certified pathologists while the remaining CSF was used for CNSide analysis (range 2-15 cm (3)). The analysis included 53 patients with breast cancer and LMD, of which 26 patients the HER2 status of the primary tumor was known, and these patients were further analyzed (**Figure 1**).

### 2.2 CNSide platform analysis

**Figure 2:**
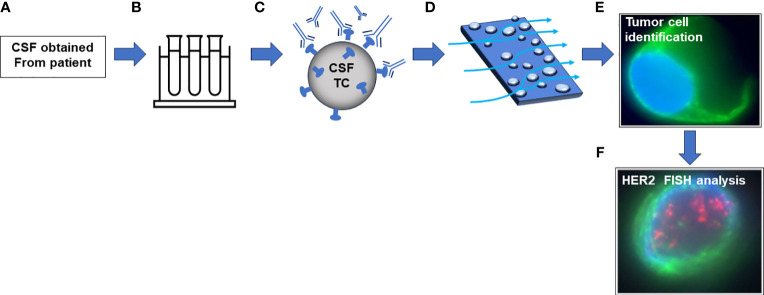
Schematic of CNSide platform. **(A)** CSF is obtained from a patient via institutional procedures. **(B)** CSF is transferred to CNSide collection tubes containing preservatives. **(C)** CSF is centrifuged, and cells are incubated with an antibody capture cocktail followed by biotinylation. **(D)** Cell suspension is incubated on a streptavidin microfluidic channel. **(E)** Cells are identified with antibody mixture containing tumor cell specific antibodies. **(F)** Tumor cells are hybridized with a probe targeting HER2.

Fresh CSF was transferred into CEE-Sure™ CSF Collection tubes (Biocept Inc, San Diego, CA) and sent to Biocept at ambient temperature for CNSide analysis. CNSide is a dual platform that allows for detection, enumeration, and biomarker analysis of CSF-TCs. CSF samples were analyzed for CSF-TC detection and HER2 characterization by FISH (**Figure 2**). The CSF-TC detection is CLIA validated and used as a commercial assay at Biocept’s CAP accredited and CLIA licensed laboratory and ordered at the physician’s discretion.

### 2.3 CSF tumor cell capture and detection

Fresh CSF samples were centrifuged, and the CSF-TC pellet was used for CNSide tumor cell enumeration. Cells are captured in the CSF via hybridization with a 10-antibody capture cocktail, followed by biotinylation. Biotinylated cells are pulled through a streptavidin coated microfluidic device, resulting in immobilization of cells. Tumor cells are then identified via immunocytochemistry analysis using specific markers, including various cytokeratin antibodies, CD45, and 4’,6’-diamindino-2-phenylindole (DAPI). Only cells that are positive for tumor associated cytokeratins and DAPI and negative for CD45 are designated CSF-TCs and included in the enumeration results as described by Pecot et al. (22).

### 2.4 HER2 FISH analysis

Cells immobilized in the microfluidic channels are hybridized with probes detecting amplification for HER2 and CEP17 (both from Abbott, Des Plaines, IL). HER2 amplification was defined by ≥ 2.0 HER2 to CEP17 ratio in one or more CSF-TC, or ≥ 6 HER2 signals per cell. The volume of CSF analyzed and total number of CSF-TCs varied per sample (due to variability in patient associated clinical factors such as tumor burden, treatment etc.). A minimum of 50 cells (and up to 100 cells) were randomly selected across the microfluidic channel and evaluated for HER2 amplification in each sample. If less than 50 cells were present in the microfluidic channel, all cells were analyzed for HER2 amplification.

## 3 Results

### 3.1 Patient characteristics and analysis

Between April 2020 and October 2022, 53 patients having breast cancer LMD who were treated at the two institutions (UT Southwestern and Northwestern University) were evaluated using CNSide. For a total of 26 unique patients, HER2 positivity of the primary tumor was known; this group was further analyzed (**Figure 1**) for HER2 status comparison between CSF and the primary tumor, and comparison of tumor cell detection between CNSide and conventional cytology (n=26 patients). CSF-TC HER2 was detected in 16 unique patients, and these were analyzed for HER2 FISH distribution analysis in the CSF. Analysis of the HER2 FISH status changes in the CSF for patients of which multiple CSF draws were analyzed sequentially during their LMD treatment was evaluated in 14 of these 26 patients (**Table 1**). Median age was 57 years (range 34 to 81 years). The median number of CSF draws was 4, ranging from 1 to 33 draws, with 12 patients having had one CSF draw and 8 patients more than 4 draws (**Table 1B**). Furthermore, in 23 patients the first draw was performed via lumbar puncture (LP), in 2 via an Ommaya intracranial reservoir device and in 1 patient CSF was collected during surgery. For the subsequent draws, one patient had an LP as well as an Ommaya draw, for 5 patients only LPs were performed and for 7 patients only Ommaya reservoir taps were performed, for one patient the CSF draw modality was unknown (**Table 1C**).

**Table 1:** Patient characteristics and CSF draw information.

A. Patient characteristics	
	N (unique patients)=26
Age range	34-81
ER/PR/HER2 positive	2
ER+/PR+/HER2 negative	6
ER+/PR-/HER2+	1
ER+/PR-/HER2-	4
ER+/PR-/HER2 Equivocal	2
ER-/PR+/HER2-	0
ER-/PR-/HER2+	1
ER/PR/HER2 negative	5
ER-/PR-/HER2 Equivocal	1
ER, PR or HER2 status unknown	4

**Table:** 

B. Number of CSF draws per patient	
Number of Patients	Number of serial draws
12	1
4	2
2	3
3	4
1	7
1	10
1	12
1	15
1	33

**Table:** 

C. Modalities of CSF draw						
	LP	Ommaya	Intraoperative	Unknown	LP and Ommaya	Total number of patients
First CSF Draw	23	2	1	0	0	26
Subsequent CSF Draws	5	7	0	1	1	14

### 3.2 HER2 discordance between primary tumor and LMD: the HER2 flip

**Figure 3:**
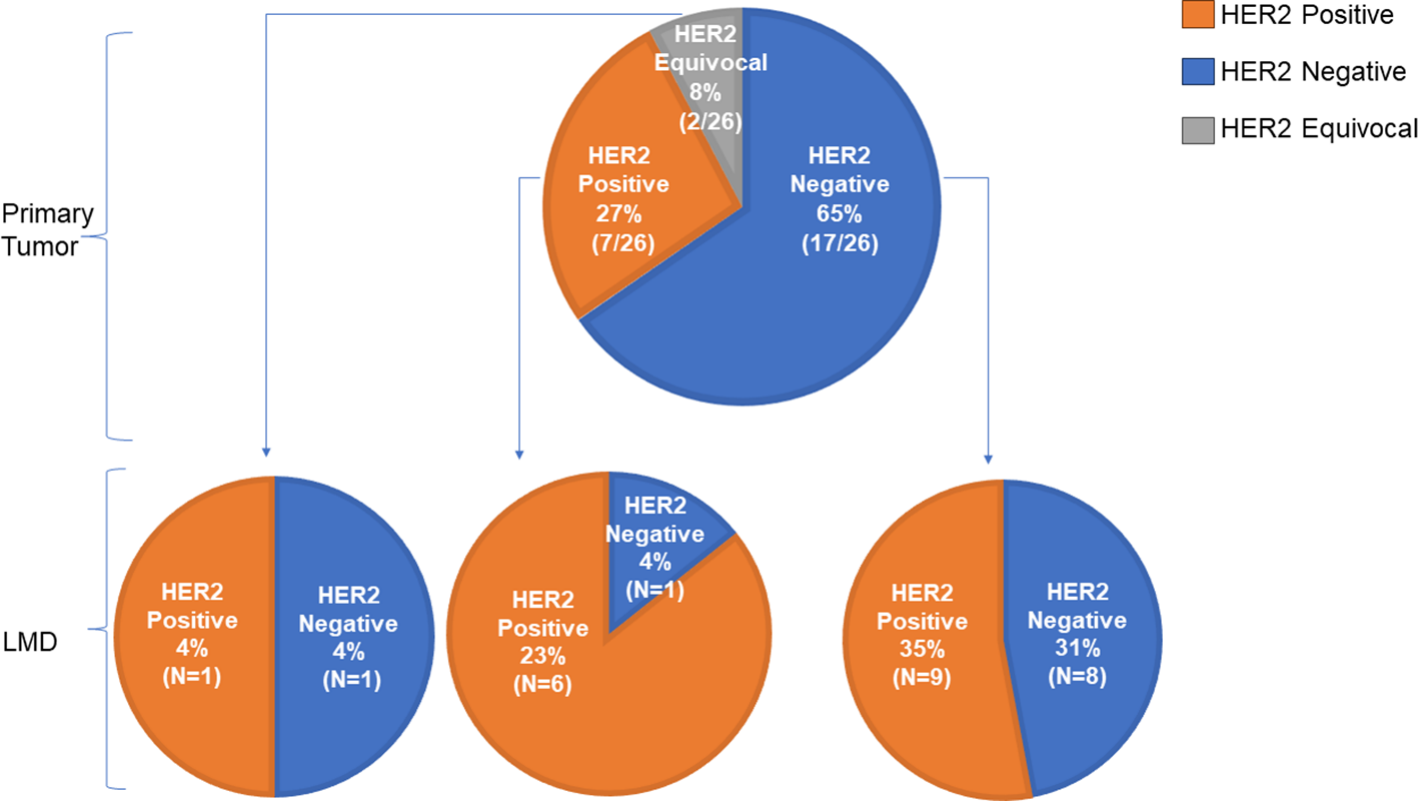
Comparison of HER2 status between primary tumor and LMD. Of 26 patients, the HER2 status of the primary tumor was compared to that of the LMD. A ‘flip’ in the HER2 status between the primary tumor and LMD was observed in 38% (10/26) patients having a HER2 positive or negative primary tumor. For 35% (9/26) patients, the primary tumor was HER2 negative and the LMD was HER2 positive.

For 26 patients, the HER2 status of the primary tumor (as analyzed by immunohistochemistry) was compared to that of the CSF. In this group, 27% (7/26) patients had a HER2 positive primary tumor, 65% (17/26) patients had a HER2 negative primary tumor, and 8% (2/26) had a HER2 equivocal primary tumor (**Figure 3**). A ‘flip’ in the HER2 status between the primary tumor and LMD was observed in 38% (10/26) patients having a HER2 positive or negative primary tumor. For 35% (9/26) patients, the primary tumor was HER2 negative and the LMD was HER2 positive. One patient (4%) had a HER2 positive primary tumor with a HER2 negative LMD. The two patients who had a HER2 equivocal primary tumor, had respectively a HER2 positive and HER2 negative LMD tumor (**Figure 3**).

### 3.3 Improved CSF tumor cell detection with CNSide compared to cytology

Cytology and CNSide was performed on matched CSF samples on all patients (N=26). When compared for tumor cell detection at the first CSF draw, CNSide detected tumor cells in 100% of patients, whereas cytology detected cells in 65% (17/26) of the patients. For 34% (9/26) patients, cytology was negative and CNSide was positive for tumor cells. When all individual CSF draws were compared (n=120), CNSide detected cells in 78% (96/120) of the samples, vs. 40% (48/120) by cytology. Two samples had a ‘rare atypical’ cytology result, and one of those samples was positive and one was negative for tumor cell detection by CNSide. Four samples had only CNSide performed and no cytology result.

### 3.4 HER2 characterization on CSF tumor cells by FISH analysis

**Figure 4:**
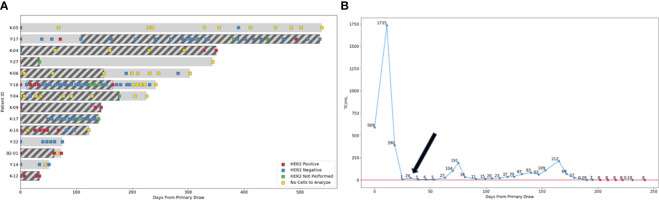
Switch in HER2 LMD status throughout therapy. **(A)** For 14 patients CSF was analyzed in sequential fashion for HER2 amplification throughout their treatment. In 29% of the patients (4/14) LMD HER2 status switched twice throughout their treatment (Patients Y-17, K-04, Y-16 and K-10). For two patients, HER2 status switched once (patient K-04 and K-10) and for two patients up to two or three switches occurred during treatment (patients Y-17 and Y-16 respectively). Two patients with HER2 negative primary tumors, where HER2 amplification was detected after LMD diagnosis (Y-17 and Y-16) had anti-HER2 therapy administered via the Ommaya reservoir added to their treatment regimen (indicated by dark diagonal bars). Four patients where HER2 amplification in the CSF was detected at diagnosis had anti-HER2 targeted therapy added to their LMD directed therapy from the start of treatment (K-04, K-10, B2-01 and K-12). Squares indicate the time points when CSF was analyzed by CNSide; Red square- HER2 positive CSF, blue square- HER2 negative CSF, green square- HER2 FISH not performed, yellow square- no cells in the CSF were detected, so no HER2 FISH result was obtainable. **(B)** LMD breast cancer patient Y-17 with a HER2 negative primary tumor shows positive clinical response to anti-HER2 directed therapy after HER2 amplification was detected in the LMD tumor (black arrow), as indicated by the CSF tumor cell numbers remaining well below that detected at diagnosis, in combination with clinical evaluation and MRI (not shown). Blue squares: sequential time points where CSF was analyzed by CNSide, red squares: time points where CNSide did not detect tumor cells. The number of cells is normalized to volume CSF drawn for a given time point, and is indicated above each square.

For the 16 patients where HER2 amplification was detected in the CSF (**Figure 1**), additional information on the HER2 amplification results of the CSF-TCs including the number of HER2 positive cells detected and the average number of HER2 signal per cell is shown in **Supplementary Figure 1**. The number of cells evaluated ranged from 1 to 100 cells per patient and was dependent on the number of cells present in the sample. The median number of HER2 amplified cells detected was 3, ranging between 1 and 100 amplified cells across the 16 patients, with 75% (12/16) of the patients having between 1 and 10 HER2 amplified cells and 25% (4/16) of the patients having more than 11 HER2 amplified cells (**Figure 4A**). The median average of HER2 copies per cell detected was 6, ranging between 2 and 25 copies (**Figure 4B**). On average, the median HER2/CEP17 ratio was 2, ranging between 2 and 25 (**Supplementary Figure 1**).

### 3.5 HER2 status of the LMD tumor can change during therapy

For the 14 patients where multiple serial CSF analysis in sequential fashion during treatment was performed, the HER2 status of the LMD tumor was compared between different time points (**Figure 4**). In 29% of the patients (4/14) LMD HER2 status switched throughout their treatment (Patients Y-17, K-04, Y-16 and K-10, **Figure 4A**). For two patients, HER2 status switched once (patient K-04 and K-10), whereas two or three switches occurred during treatment for the other two patients (patient Y-17 and Y-16 respectively). For these two patients, anti-HER2 therapy was administered via the Ommaya reservoir upon detection of HER2 amplification in the CSF (indicated by dark diagonal bars). Four patients where HER2 amplification in the CSF was detected at diagnosis had anti-HER2 targeted therapy added to their LMD directed therapy from the start of treatment (K-04, K-10, B2-01 and K-12). CNSide provided tumor cell counts at each CSF evaluation. Previously, it was suggested that changes in CSF tumor cell counts can be a measure of the response of the LMD tumor to therapy (21). This observation was replicated in analysis of the patients described here. Furthermore, it was noted that anti-HER2 directed therapy given based on the HER2 status of the LMD tumor reduced the number of CSF tumor cells, which was associated with a positive clinical response to therapy (assessed via MRI, cytology and clinical evaluation). This is exemplified by patient Y-16 who had a HER2 negative primary tumor and HER2 amplification on the CSF tumor cells at the time of LMD diagnosis. At LMD diagnosis in this patient, CNSide detected 589 cells/ml. Treatment commenced and consisted of IT topotecan and trastuzumab. Within approximately one month, tumor cells decreased 84-fold to 7 cells/ml, together with disappearance of HER2 positive CSF tumor cells within the following two weeks (**Figures 4A, B**). No HER2 amplification on CSF tumor cells was observed for the next 4 months, and clinically the patient responded well to therapy. The CSF-TC density also remained well below the values observed at diagnosis, ranging between 7 and 212 cells/ml during that time.

## 4 Discussion

The current standard of care for the diagnosis and response measurement in LMD is laden with challenges. The “gold standard” in LMD diagnosis is CSF cytology, however this modality has poor sensitivity and does not allow for quantification of tumor cells. Radiographic findings are limited in LMD and are not always present; clinical findings can be confounded by many other variables. In recent years, technology used for circulating tumor cell and ctDNA analysis in serum is being adapted for use in CSF. First generation iterations of this (i.e. CellSearch) showed improved sensitivity in testing as well as the ability to quantify tumor cells in the CSF and follow serially. Newer generation CSF testing in these domains, such as CNSide testing expand beyond epithelial cell detection, also allow quantification of increasing cell quantities, and include the key epithelial to mesenchymal transition cells seen in LMD. This IRB approved retrospective protocol aimed to assess the impact of this testing in patients with LMD. Through this aggregate data as well as deidentified patient cases, this retrospective review demonstrated multiple key points.

### 4.1 CNSide testing can detect CSF-TCs not seen on traditional CSF cytology

CSF cytology has been known to have poor sensitivity. With a single CSF draw, CSF cytology sensitivity is thought to be approximately 60%. This is similar to what we found in this retrospective analysis with only 65% of the analyzed patients demonstrating positive cytology, whereas CNSide detected CTCs in 100% in these patients with clinical suspicion of LMD based on radiographic findings or examination findings. CNSide’s better-suited ability to detect CSF-TC indicative of LMD could lead to earlier diagnosis, more expedient intervention, and potentially better outcomes for these patients.

### 4.2 HER2 status can be different in the CSF as compared to primary tumor/other systemic metastases

This concept has previously been demonstrated comparing systemic metastases vs brain parenchymal metastases (16), however has not yet been shown on this scale with CSF testing as demonstrated herein. Furthermore, CSF collection and HER2 status testing is less invasive and less risky than brain tissue biopsy which requires surgical intervention. HER2 positivity detection in CSF may open additional doors for treatment options available to each patient. Anti-HER2 therapeutic agents are modalities that have shown favorable results and can positively impact both a patient’s quality and quantity of life (13).

### 4.3 Using the CNSide platform, CSF-TC trends can be followed to assess for treatment response and improve treatment decisions

This CSF diagnosis pattern has the unique ability to enumerate tumor cells in the CSF, a key component missing with CSF cytology. This ability allows for improved treatment monitoring both in treatment response assessment and in monitoring for recurrence. Additionally, trends may be followed in HER2 positivity with gain or loss of HER2 amplification across disease trajectory or treatment, which will support treatment decision making along the course of LMD treatment.

Despite these merits, there are multiple shortcomings to this study. First and foremost, this is a retrospective analysis which confers potential selection or misclassification bias. Because of the retrospective nature of this work, not all datapoints were available in all included patients. Additionally, data collection timepoints were not uniform across sites and patients. Much of these biases will be overcome with the results of the prospective FORESEE study which started enrollment in March 2023 (NCT05414123). This prospective study enrolled patients with lung or breast cancer with suspected LMD and took concurrent timepoints of radiographic, clinical, cytologic and CNSide testing for simultaneous comparison. The study is currently closed to accrual following enrollment of the planned 40 patients however is currently suspended due to financial insolvency of the company. The results of this prospective study are pending and planned to be published.

## 5 Conclusion

Overall, this study posits the importance of measuring tumor markers in the CSF such as HER2. We have captured a significant rate of this “HER2 flip” in this multi-institutional retrospective study, which is the first time this finding has been published on this scale in CSF/LMD literature. Furthermore, we highlight the importance of serial quantification of CSF-TCs in both diagnosis and treatment management, particularly while on LMD directed therapy. Given their ability to more effectively detect CSF-TC for early LMD diagnosis, to detect LMD mutations discordant to primary tumor for treatment, and to reliably serially follow expression of these mutations to modify treatment strategies, CSF-TC testing modalities such as CNSide are an essential target for further investigation and optimization in order to improve outcomes in this fragile patient population.

## Data availability statement

The raw data supporting the conclusions of this article will be made available by the authors, without undue reservation.

## Ethics statement

The studies involving humans were approved by Northwestern University Institutional Review Board, affiliated with Northwestern University and University of Texas Southwestern Medical Center Institutional Review Board, affiliated with University of Texas Southwestern.

## Author contributions

PK: Conceptualization, Data curation, Formal analysis, Investigation, Methodology, Supervision, Writing – original draft, Writing – review & editing. BB: Formal analysis, Investigation, Visualization, Writing – original draft, Writing – review & editing. PC: Data curation, Formal analysis, Methodology, Visualization, Writing – review & editing. SN: Methodology, Supervision, Writing – review & editing. AT: Methodology, Writing – original draft, Writing – review & editing. DP: Writing – review & editing. MY: Data curation, Methodology, Supervision, Writing – review & editing.

## Supplementary material

Supplementary Figure 1 CSF HER2 amplification analysis of LMD breast cancer patients. **(A)** For 16 patients, between 1 and 100 CSF tumor cells were evaluated for HER2 amplification (blue bars), of which the median number of cells amplified was 3 ranging between 1 and 100 cells. **(B)** The median average number of HER2 copies was 6, ranging between 2.4 and 25 copies per cell. **(C)** The median average ratio HER2/CEP17 was 2, ranging from 0.89 to approximately 12 per cell.

## References

[1] Remon J Le Rhun E Besse B. Leptomeningeal carcinomatosis in non-small cell lung cancer patients: A continuing challenge in the personalized treatment era. Cancer Treat Rev. (2017) 53:128–37. doi: 10.1016/j.ctrv.2016.12.006 28110254

[2] Franzoi MA Rosa DD Zaffaroni F Cronemberger E Queiroz GS Cordeiro de Lima V. Advanced stage at diagnosis and worse clinicopathologic features in young women with breast cancer in Brazil: A subanalysis of the AMAZONA III study (GBECAM 0115). Cancer Res. (2019) 79:P1–08–27. doi: 10.1158/1538-7445. SABCS 18-P1-08-27 PMC6882517 31730380

[3] Mittica G Senetta R Richiardi L Ruda R. Coda R. Castaellano A.. Meningeal carcinomatosis underdiagnosis and overestimation: incidence in a large consecutive and unselected population of breast cancer patients. BMC Cancer. (2015) 15:1021. doi: 10.1186/s12885-015-2042-y 26715407 PMC4696095

[4] Boogerd W Hart AA van der Sande JJ Engelsman E. Meningeal carcinomatosis in breast cancer. Prognostic factors and influence of treatment. Cancer. (1991) 67:1685–95. doi: 10.1002/(ISSN)1097-0142 2001559

[5] Wijetunga NA Boire A Young RJ Yamada Y. Wolden S. Yu H.. Quantitative cerebrospinal fluid circulating tumor cells are a potential biomarker of response for proton craniospinal irradiation for leptomeningeal metastasis. Neuro-Oncol. Adv. (2021) 3:vdab181. doi: 10.1093/noajnl/vdab181 PMC8717892 34993483

[6] Diaz M Singh P Kotchetkov IS Skakodub A. Meng A. Tamer C.. Quantitative assessment of circulating tumor cells in cerebrospinal fluid as a clinical tool to predict survival in leptomeningeal metastases. J Neurooncol. (2022) 157:81–90. doi: 10.1007/s11060-022-03949-1 35113288 PMC9119011

[7] Yang JCH Kim SW Kim DW Lee JS. Cho BC. Ahn JS.. Osimertinib in patients with epidermal growth factor receptor mutation–positive non–small-cell lung cancer and leptomeningeal metastases: the BLOOM study. J Clin Oncol. (2020) 38:538–47. doi: 10.1200/JCO.19.00457 PMC7030895 31809241

[8] Le Rhun E Preusser M van den Bent M Andratschke N Weller M. How we treat patients with leptomeningeal metastases. ESMO Open. (2019) 4:e000507. doi: 10.1136/esmoopen-2019-000507 31231573 PMC6555600

[9] Kumthekar P Le Rhun E. Brain metastases and leptomeningeal disease. Contin Minneap Minn. (2023) 29:1727–51. doi: 10.1212/CON.0000000000001354 38085896

[10] Novel approaches to treatment of leptomeningeal metastases: Current Opinion in Neurology. Available online at: https://journals.lww.com/co-neurology/fulltext/2023/12000/novel_approaches_to_treatment_of_leptomeningeal.15.aspx (Accessed February 19, 2024). 10.1097/WCO.0000000000001218 37865856

[11] Murthy RK O’Brien B Berry DA Singareeka-Raghavendra A. Monroe MG. Johnson J.. Abstract PD4-02: Safety and efficacy of a tucatinib-trastuzumab-capecitabine regimen for treatment of leptomeningeal metastasis (LM) in HER2-positive breast cancer: Results from TBCRC049, a phase 2 non-randomized study. Cancer Res. (2022) 82:PD4–02. doi: 10.1158/1538-7445.SABCS21-PD4-02

[12] Niikura N Yamanaka T Nomura H Shiraishi K. Kusama H Yamamoto M.. Treatment with trastuzumab deruxtecan in patients with HER2-positive breast cancer and brain metastases and/or leptomeningeal disease (ROSET-BM). NPJ Breast Cancer. (2023) 9:1–8. doi: 10.1038/s41523-023-00584-5 37821514 PMC10567705

[13] Kumthekar PU Avram MJ Lassman AB Lin NU. Lee E. Grimm SA.. A phase I/II study of intrathecal trastuzumab in human epidermal growth factor receptor 2-positive (HER2-positive) cancer with leptomeningeal metastases: Safety, efficacy, and cerebrospinal fluid pharmacokinetics. Neuro-Oncol. (2023) 25:557–65. doi: 10.1093/neuonc/noac195 PMC10013631 35948282

[14] Gennari A André F Barrios CH Cortes J. De Azambuja E. DeMichele A.. ESMO Clinical Practice Guideline for the diagnosis, staging and treatment of patients with metastatic breast cancer☆. Ann Oncol. (2021) 32:1475–95. doi: 10.1016/j.annonc.2021.09.019 34678411

[15] Gradishar WJ Moran MS Abraham J Abramson V. Aft R. Agnese D.. NCCN guidelines^®^ Insights: breast cancer, version 4.2023: featured updates to the NCCN guidelines. J Natl Compr Canc. Netw. (2023) 21:594–608. doi: 10.6004/jnccn.2023.0031 37308117

[16] Brastianos PK Carter SL Santagata S Cahill DP. Taylor-Weiner A. Jones RT.. Genomic characterization of brain metastases reveals branched evolution and potential therapeutic targets. Cancer Discovery. (2015) 5:1164–77. doi: 10.1158/2159-8290.CD-15-0369 PMC4916970 26410082

[17] Lin X Fleisher M Rosenblum M Lin O. Boire A. Briggs S.. Cerebrospinal fluid circulating tumor cells: a novel tool to diagnose leptomeningeal metastases from epithelial tumors. Neuro-Oncol. (2017) 19:1248–54. doi: 10.1093/neuonc/nox066 PMC5570249 28821205

[18] van Bussel MTJ Pluim D Milojkovic Kerklaan B Bol M. Sikorska K. Linders DTC.. Circulating epithelial tumor cell analysis in CSF in patients with leptomeningeal metastases. Neurology. (2020) 94:e521–8. doi: 10.1212/WNL.0000000000008751 31907288

[19] Malani R Fleisher M Kumthekar P Lin X. Omuro A. Groves MD.. Cerebrospinal fluid circulating tumor cells as a quantifiable measurement of leptomeningeal metastases in patients with HER2 positive cancer. J Neurooncol. (2020) 148:599–606. doi: 10.1007/s11060-020-03555-z 32506369 PMC7438284

[20] Margarido AS Uceda-Castro R Hahn K De Bruijn R. Kester L. Hofland I.. Epithelial-to-mesenchymal transition drives invasiveness of breast cancer brain metastases. Cancers. (2022) 14:3115. doi: 10.3390/cancers14133115 35804890 PMC9264851

[21] Puri S Malani R Chalmers A Kerrigan K. Patel SB. Monynahan K.. Keeping a track on leptomeningeal disease in non–small cell lung cancer: A single-institution experience with CNSideTM. Neuro-Oncol. Adv. (2023) 6:vdad150. doi: 10.1093/noajnl/vdad150 PMC10776200 38196737

[22] Pecot CV Bischoff FZ Mayer JA Wong KL. Pham T Bottsford-Miller J.. A novel platform for detection of CK+ and CK– CTCs. Cancer Discovery. (2011) 1:580–6. doi: 10.1158/2159-8290.CD-11-0215 PMC3237635 22180853

