# Supplementary figures and images for: The HER2 flip-HER2 amplification of tumor cells in the cerebrospinal fluid of breast cancer patients with leptomeningeal disease: implications for treating the LM tumor with anti-HER2 therapy
Source: Front Oncol. 2024 May 17;14:1402651. doi: 10.3389/fonc.2024.1402651 (PMC11140729; doi:10.3389/fonc.2024.1402651)

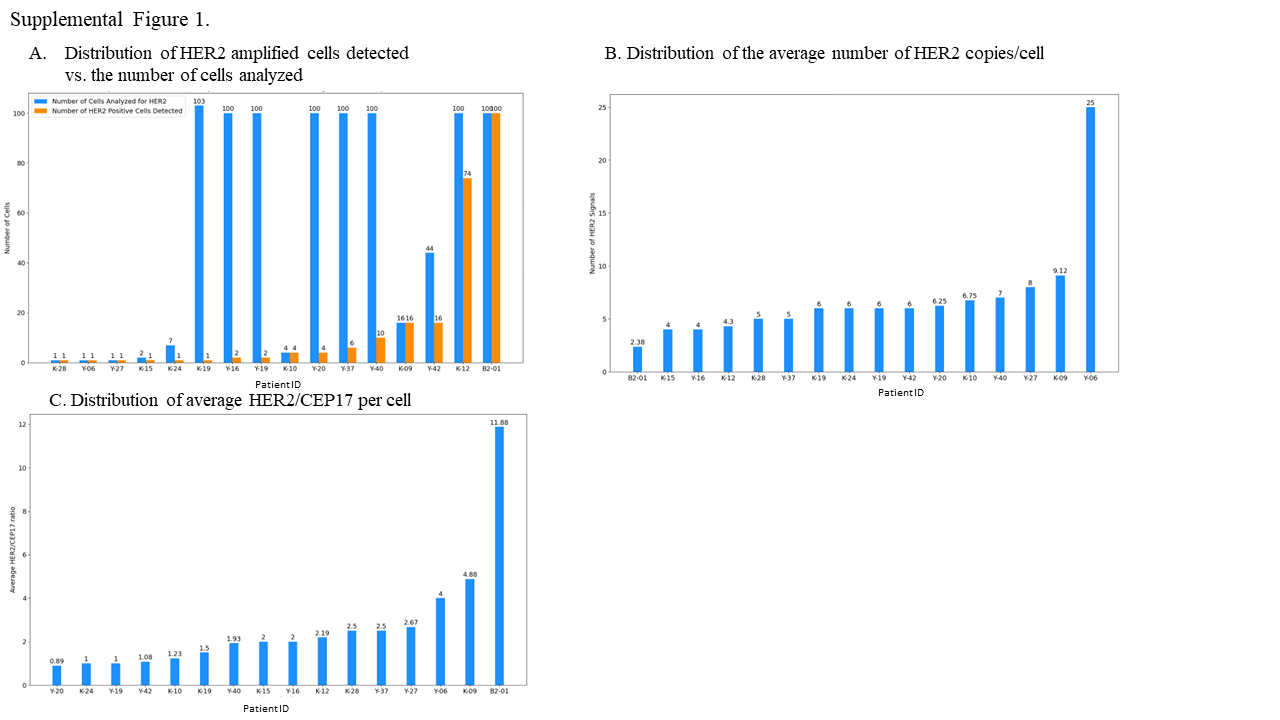

Supplement: Supplementary Figure 1 — CSF HER2 amplification analysis of LMD breast cancer patients. (A) For 16 patients, between 1 and 100 CSF tumor cells were evaluated for HER2 amplification (blue bars), of which the median number of cells amplified was 3 ranging between 1 and 100 cells. (B) The median average number of HER2 copies was 6, ranging between 2.4 and 25 copies per cell. (C) The median average ratio HER2/CEP17 was 2, ranging from 0.89 to approximately 12 per cell. [file Image_1.tif]
